# Quantifying Cognitive Impairment After Sleep Deprivation at Different Times of Day: A Proof of Concept Using Ultra-Short Smartphone-Based Tests

**DOI:** 10.3389/fnbeh.2021.666146

**Published:** 2021-04-13

**Authors:** Benjamin C. Holding, Michael Ingre, Predrag Petrovic, Tina Sundelin, John Axelsson

**Affiliations:** ^1^Department of Clinical Neuroscience, Karolinska Institutet, Stockholm, Sweden; ^2^Department of Sociology, University of Copenhagen, Copenhagen, Denmark; ^3^Department of Psychology, Stockholm University, Stockholm, Sweden

**Keywords:** sleep, sleep deprivation, executive function, memory, smartphone applications, neuropsychological tests, reaction time, cognitive performance

## Abstract

Cognitive functioning is known to be impaired following sleep deprivation and to fluctuate depending on the time of day. However, most methods of assessing cognitive performance remain impractical for environments outside of the lab. This study investigated whether 2-min smartphone-based versions of commonly used cognitive tests could be used to assess the effects of sleep deprivation and time of day on diverse cognitive functions. After three nights of normal sleep, participants (*N* = 182) were randomised to either one night of sleep deprivation or a fourth night of normal sleep. Using the Karolinska WakeApp (KWA), participants completed a battery of 2-min cognitive tests, including measures of attention, arithmetic ability, episodic memory, working memory, and a Stroop test for cognitive conflict and behavioural adjustment. A baseline measurement was completed at 22:30 h, followed by three measurements the following day at approximately 08:00 h, 12:30 h, and 16:30 h. Sleep deprivation led to performance impairments in attention, arithmetic ability, episodic memory, and working memory. No effect of sleep deprivation was observed in the Stroop test. There were variations in attention and arithmetic test performance across different times of day. The effect of sleep deprivation on all cognitive tests was also found to vary at different times of day. In conclusion, this study shows that the KWA’s 2-min cognitive tests can be used to detect cognitive impairments following sleep deprivation, and fluctuations in cognitive performance relating to time of day. The results demonstrate the potential of using brief smartphone-based tasks to measure a variety of cognitive abilities within sleep and fatigue research.

## Introduction

Cognitive performance is known to be impaired following sleep deprivation (Wickens et al., 2015). This impairment is the most evident during basic cognitive tasks such as sustained attention, but can also be seen in cognitive domains such as working memory, episodic memory, and impulse inhibition (Killgore, 2010; Lim and Dinges, 2010; Wickens et al., 2015).

Cognitive performance has also been shown to vary as a function of time of day (Furnham and Rawles, 1988; Riley et al., 2017; Lewandowska et al., 2018). Individuals generally perform worse during the night and during the “post-lunch dip,” and often show increasing performance from morning to early evening (Carrier and Monk, 2000). Time-of-day effects also appear in individuals who have not slept sufficiently (Lo et al., 2012; Jarraya et al., 2014; Bougard et al., 2016). The time-of-day aspects of cognitive functioning are driven by the interaction of sleep homeostasis (time awake and previous sleep drive) and circadian rhythmicity (Dijk et al., 1992; Gabehart and Van Dongen, 2017; Deboer, 2018). To give an experimental example of how the systems interact, one study found that increased sleep pressure augmented the circadian influence on subjective alertness, sustained attention, and a number of executive functions, specifically during the morning (Lo et al., 2012). However, how these processes interact and influence cognition has not been well studied in natural environments, outside of the lab. One of the key reasons for this is the lack of suitable methods to measure cognitive performance in the field.

The rise of portable electronic devices, such as smartphones and tablets, provides new possibilities for cognitive testing outside the research lab. For example, field studies on the effect of time of day on cognitive functioning have previously been hindered by equipment and time requirements. If participants are required to repeatedly complete long computer tasks, they need to volunteer a lot of their time and have access to the computers. Early studies have shown that touchscreen versions of sustained attention tests [e.g., the psycho-motor vigilance test, PVT^1^ (Dinges and Powell, 1985)] are valid instruments for measuring reduced alertness due to sleep deprivation (Grant et al., 2017; Arsintescu et al., 2019). However, the ability of such touchscreen-based tests to assess more complex cognitive abilities has not been evaluated in relation to sleep loss and time of day.

Test-induced fatigue is also a major problem in cognitive testing, particularly for long tests or long cognitive batteries that require sustained attention. This time-on-task effect has been found for many cognitive functions, with poorer performance when tests are carried out continuously for a long time (Mackworth, 1948; Lim et al., 2010; Blain et al., 2016). These performance impairments, in addition to being the result of mental fatigue (Warm et al., 2008), may also result from decreased motivation and increased boredom (Pattyn et al., 2008; Möckel et al., 2015). Meanwhile, shorter attention tests, of 3–5 min, have near equal validity for measuring attention compared to longer tests (Roach et al., 2006; Basner et al., 2011), indicating the possibility of measuring cognitive performance while reducing the risk of the time-on-task effect. Other shorter tests, such as 2-min math tests, have also been shown to be sensitive to diurnal variation in laboratory conditions (e.g., Wertz et al., 2006). However, there is a lack of shorter tests that can be used outside the laboratory, particularly for measuring several different cognitive functions.

In this study, we aimed to assess whether short 2-min cognitive tests could be used toquantify the effects of sleep deprivation and time of day on changes in performance in several cognitive functions, as well as investigate potential interactions between these. Using the recently developed “Karolinska WakeApp”—a brief mobile application for assessing several core cognitive functions—we measured simple attention, arithmetic ability, episodic memory, working memory, and cognitive conflict and behavioural adjustment on a Strooptest. The cognitive tests were based on existing validated tasks used in multiple areas of research.

Based on previous findings (Killgore, 2010; Lim and Dinges, 2010; Wickens et al., 2015), we hypothesised that all cognitive functions would be negatively affected by sleep loss, with the largest effects seen in the basic functions, such as simple attention, and smaller effects in complex functions such as arithmetic ability and Stroop performance. We also expected to see a general increase in reaction-time variability and lapses following sleep deprivation, due to state instability (Doran et al., 2001). Regarding the effect of time of day on cognitive performance, analyses were more exploratory, with the tentative hypothesis that performance would generally be worse in the morning, with gradual improvement throughout the day (Carrier and Monk, 2000). Since there is no clear consensus on whether time of day would be expected to moderate the effect of sleep loss (Deboer, 2018), especially in different cognitive domains, we analysed these effects in an exploratory manner.

## Materials and Methods

### Participants

One-hundred and eighty-two healthy individuals (age range 18–45, 103 women) participated in this randomised between-subjects design experiment. Ninety-one participants (mean age = 25.4, age SD = 6.2, 52 women) were randomised to the sleep deprivation condition and 91 participants (mean age = 25.3, age SD = 6.8, 51 women) to the normal-sleep condition. Potential participants were screened through an online questionnaire to exclude those with physical and mental health problems, including clinical sleep disorders, or poor habitual sleep. All participants reported a sleep need of 7–9 h per night, and in the prior 3 weeks had not visited a country three or more time zones away. A complete list of screening criteria can be found in a previous paper by the group (Holding et al., 2019b). No significant difference in chronotype (“Are you a morning or an evening person?” 1-very much morning, 5-very much evening) between conditions was observed (mean well-rested = 3.22, mean sleep deprived = 2.95, *p* = 0.10). The study was approved by the Stockholm Regional Ethical Review Board (no. 2014/1766-32) and participants received financial compensation.

### Measures

#### Karolinska WakeApp

The Karolinska WakeApp (KWA) is a smartphone-based cognitive test battery conducted on the participant’s own phone. The application runs through a web-browser and has been developed to run safely on Safari for iPhones and Chrome for Androids. The majority of participants used iPhones, and iPhones were available for participants to borrow if they did not wish to use, or have access to, an own smartphone. However, we made no restrictions on what smartphones participants could use. The KWA consists of five separate cognitive tests, specified below. Each test is approximately 2 min long, although two of them have a self-paced component. Example images of each test can be found in Supplementary Figures 1–6.

Distributions of response times for the cognitive tests with a response-time element can be seen in Supplementary Figure 7. Note that smartphone touchscreens include a latency between the time of pressing the screen and the response being logged (in the case of the KWA the touch response needs to be logged by the web browser; Henze et al., 2016; Arsintescu et al., 2017). The latency means that the response times recorded are systematically longer than the true response times. While this does not impact performance comparisons between or within participants measured with the KWA, it means that response times vary compared to other task formats (e.g., tasks requiring a mechanical button press or touchscreen tasks where the latency has been accounted for within the application). In this study, we did not make any correction for response time latency since it was not measured.

##### Simple Attention

A single choice reaction time test similar to a PVT. Participants were required to click on a blue button displayed on the lower half of the mobile phone touchscreen as soon as a cue was presented (the letter “p”) in the upper half. The inter-stimulus intervals varied randomly from 2 to 5 s. We analysed performance in terms of response time (RT), response time variability (intra-individual standard deviation, RTV), and percentage of responses that were classified as lapses. Due to the increased response times recorded in the KWA because of touchscreen latency, the appropriate threshold of setting a lapse could not be the same as that used by the classic PVT (typically >500 ms; Dinges and Powell, 1985). Instead, we used a threshold of >1,000 ms to signify a lapse. This represents approximately double the observed mean RT (506 ms) which has been previously suggested to be an appropriate operationalisation of a lapse as well as used in previous studies (Basner and Dinges, 2011; Rajaraman et al., 2012; Zhang et al., 2012). However, it is possible that lapses measured in this study are a conservative measurement and other thresholds may be more sensitive to the effects of sleep loss or fatigue. Responses that were under 100 ms or over 3,000 ms were removed.

##### Arithmetic Ability

Participants were presented with simple arithmetical addition questions and required to calculate the answer and type it into the phone self-paced. Such addition tests have previously been used to measure cognitive throughput (Jewett et al., 1999; Hofer-Tinguely et al., 2005). Participants were asked to respond as quickly and correctly as possible, with new questions presented after every response until the time ran out. Performance was measured in terms of probability of making a mistake and the speed of each response (in milliseconds). Responses that were under 100 ms or over 10,000 ms (allowing sufficient time for mental calculations) were removed.

##### Episodic Memory

Participants were asked to remember a list of 12 words which was presented for 12 s. A fixation cross then appeared for 5 s. Following this, participants were shown a list of 24 words, containing the original 12 words as well as 12 dummy words, and asked whether each word was previously shown (yes/no). After a 5-s fixation cross, the original 12 words were presented with 12 new dummy words, and the participant was again asked using forced choice whether each word was previously shown. Performance was assessed as the probability of misremembering each word as being present or absent in the original word list. This self-paced test is based on the Claeson–Dahl test (Sandström et al., 2005).

##### Working Memory

Based on a spatial working memory test (Fry and Hale, 1996; Klingberg et al., 2002), participants were presented with a 4 × 4 grid and asked to remember the sequence in which certain squares became red. Seven red squares were presented sequentially per trial. Following a fixation-cross for 1 s, the app gave a suggestion about at what point in the seven-square presentation a particular square became red. To answer, the subjects had to decide whether that particular square had become red, and if yes, whether it was in that exact order in the sequence. Participants made a forced choice response to whether the suggestion was true or false by clicking the appropriate button. When participants completed 10 trials, performance was measured in terms of the number of mistakes made. Responses that were under 100 ms and over 10,000 ms were removed.

##### Stroop

In this Stroop colour-word test (Stroop, 1935) participants were presented with colour words, written in a coloured font. Beneath the word were four buttons (all the same colour, different words only), each representing a colour. Participants were asked to click on the button that represented the colour of the font, but not the meaning of the word (e.g., the colour of the font may be blue while the actual word was “red”). The included colours for both words and fonts were blue, red, yellow, and green. The Stroop test is an executive function test that measures cognitive conflict caused by incongruent information and its resolution. Trials showing the same colour and word meaning are defined as congruent, while trials with non-matching colour and word meanings are defined as incongruent. Each stimulus type (congruent/incongruent) can be preceded by the same or by a different stimulus type (congruent/incongruent). Therefore, each response belongs to one of the four conditions, representing the change from previous to current stimulus type: (1) incongruent-Congruent (iC); (2) congruent-Congruent (cC); (3) congruent-Incongruent (cI); and (4) incongruent-Incongruent (iI).

Our primary interests were the effects of sleep deprivation on the response to conflicting information, called *cognitive conflict*, and the ability to update one’s strategy in relation to such information, called *behavioural adjustment* (Mansouri et al., 2009). These are both important for executive control adjustments. Our measure of the effect of cognitive conflict was defined as the difference in RT between a cI trial (involving the largest amount of conflict) and an immediately preceding cC trial (involving the smallest amount of conflict). Our measure of the effect of behavioural adjustment was defined as the difference in RT for an iI trial (where a change in strategy has been implemented) compared to an immediately preceding cI trial (stimuli needing an update in strategy to optimise responding). In addition to the effects of cognitive conflict and behavioural adjustment on RT, we measured the variation in these effects as RTV. We also assessed whether the overall error rate was predicted by sleep deprivation. Responses that were under 500 ms and over 3,000 ms were removed.

#### Karolinska Sleepiness Scale

After each cognitive test (i.e., five times during each testing session), participants reported their subjective sleepiness using the single-item Karolinska Sleepiness Scale (KSS; Åkerstedt and Gillberg, 1990). Responses ranged between 1 (very alert) and 9 (very sleepy).

### Procedure

For 3 days before the test day, participants were instructed to spend 8–9 h in bed each night, turning off the light at 23:00 h ± 60 min and getting up at 07:00 h ± 60 min. To ensure compliance, participants kept a sleep diary and wore an actigraph (GeneActiv Sleep, Activinsights Limited, Kimbolton, UK). Participants were asked to avoid naps, abstain from alcohol, and not drink caffeinated drinks later than the morning of the day prior to testing (penultimate day).

To reduce learning effects, participants practiced all tests twice, once with a research assistant at an initial meeting (approximately 3–4 days prior to testing) and a second time independently at any point before 20:00 h of the penultimate day.

At midday on the penultimate day, participants were informed which condition they were placed into. This was quasi-randomised for all participants, keeping an equal number of participants within each group. Those in the sleep-deprivation condition were required to come to the lab at 22:00 h that night, and those in the control condition were instructed to continue with the same sleeping instructionas the previous days and instead arrive at 10:00 h the following day.

During sleep deprivation, participants stayed in a light-controlled lab and were free to choose their activities (e.g., watch a film, read, or use the computer). A research assistant was with the participant at all times to ensure that they stayed awake. Low-sugar food was provided if the participant was hungry. Participants in the sleep deprivation condition also took a morning walk to reduce the confounding effect of increased movement and light experienced by the well-rested condition on their commute to the lab from home.

The KWA was completed on four occasions—22:30 h (representing a “baseline” measurement point with both conditions having similar sleep history, similar time awake, and being in a similar circadian phase), 08:00 h, 12:30 h, and 16:30 h. These test-day times were chosen to be roughly equally spaced out in time, representing a morning, midday, and afternoon session. We used the measurement at 22:30 h as a point where performance was compared against the different sessions the following day. Thus, the 22:30 h “baseline” measure does not represent “peak” cognitive performance in participants, due to both circadian and homeostatic influences increasing sleepiness. Nonetheless, it is a point where performance should be comparable between the two conditions. Participants in the sleep deprivation condition, were reminded by the research assistant to complete the KWA at 22:30 h and at 08:00 h. Control participants (sleeping at home) were instructed to complete the KWA in a quiet place before they went to bed and again when they woke up. When in the lab, participants completed the KWA ina private room. During the test day, participants completed multiple other tests (all between 10:00 h and 17:30 h), the results of some of which have been published previously (e.g., Holding et al., 2017, 2019a,b). Lunch for all participants was provided at 12:00 h and a lighter snack was available at 15:45 h.

### Analytic Strategy

We took a model comparison approach to analysing the data. This reduces the risk of over-fitting the data which can lead to spurious effect estimates and allows for independent assessment of the statistical significance of fixed-effect predictors (Vandekerckhove et al., 2014; Rouder et al., 2016; Meteyard and Davies, 2020). Linear mixed-effect models were used to estimate the effect of sleep condition (sleep-deprivation or sleep-control), session (08:00, 12:30, and 16:00 h), and a potential interaction among these factors. Using R with the lme4 (Bates et al., 2014) package, a stepwise series of model comparisons were conducted for each cognitive outcome to determinethe complexity of thefinal models used to assess the effect estimates of each predictor. We also included a dummy variable representing differences in condition at 22:30 h to account for potential systematic differences in cognitive performance between conditions unrelated to sleep loss or time of day.

In the model comparisons stage, there were four levels of comparison: an intercept-only model, a base model (containing baseline condition, session, and the stimuli presentation order), a sleep-deprivation model (containing sleep condition as a dummy variable, on top of the same predictors as the base model), and an interaction model (containing a condition*session interaction, on top of the same predictors as the sleep-deprivation model). The effect estimates of the best fitting models following this process, as determined by significant differences between models in likelihood-ratio tests, are provided in the tables below. The results of the model comparisons are available in the Supplementary Material.

## Results

### Prior Sleep, Test Timing, and Test-Related Descriptive Statistics

During the three nights prior to the final experimental night, average sleep duration was similar for both conditions [well-rested = 7 h:50 min (SD = 51 min), sleep deprived = 7 h:52 min (SD = 55 min)]. On the penultimate night, the well-rested group slept an average of 7 h:50 min (SD = 55 min; mean sleep onset time = 23:42 h, mean wake time = 07:34 h) and the sleep deprivation group slept an average of 7 h:42 min (SD = 54 min; mean sleep onset time = 23:43 h, mean wake time = 07:24 h). On the final night, the well-rested group, participants slept an average of 7 h:51 min (SD = 54 min; mean sleep onset time = 23:45 h, mean wake time = 07:37 h) while the sleep deprivation group did not sleep. There were some differences in test timing between the two conditions, with the baseline evening session occurring on average 9 min earlier in the sleep deprived condition and the morning session occurring on average 48 min earlier in the sleep deprived condition (see Supplementary Table 1). Histograms of raw response times from the attention, arithmetic, working memory, and Stroop tests can be seen in Supplementary Figure 7. For raw responses in the Stroop test, response times were significantly longer for in congruent stimuli (mean = 1,174.84 ms) than for congruent (1,034.40 ms), *t*_(63,557)_ = 49.29, *p* < 0.001.

### Simple Attention

**Figure 1:**
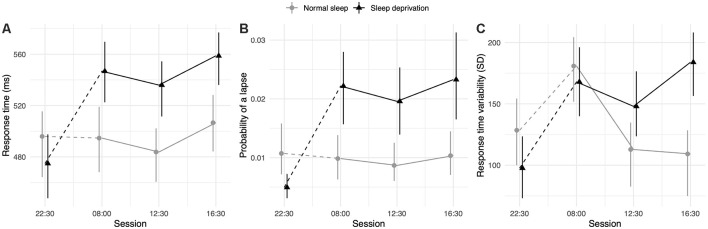
Simple attention test performance depending on sleep condition and time of day. **(A)** Represents performance measured as response time (RT; in ms). **(B)** Represents performance measured by the probability of a lapse (lapse = response >1,000 ms). **(C)** Represents performance measured as RTV (standard deviation of response time in ms). Dots represent fixed-effect estimates and error bars represent bootstrapped confidence intervals with 100 iterations. The dotted line between 22:30 h and 08:00 h represents the night of total sleep deprivation for the sleep-deprivation group.

The sleep deprivation condition showed a significant increase in RT, increased odds of lapsing, and increased RTV. Time-of-day did not significantly predict RT, but RTV was higher at 08:00 h compared to baseline (22:30 h). The effect of sleep deprivation on RTV was highest in the afternoon session (16:30 h) and significantly less in the morning (08:00 h) and lunch (12:30 h). The full results regarding simple attention can be found in Table 1, and visualisations of the predicted effects observed in the models can be found in Figure 1. The results of the model comparisons can be found in Supplementary Tables 2–4.

**Table 1:** Simple attention test performance predictions based on sleep condition and time-of-day in the final mixed-effects model.

	RT			Lapse			RTV		
*Fixed-effect predictors*	*Estimates*	*CI*	*p*	*Odds ratios*	*CI*	*p*	*Estimates*	*CI*	*p*
(Intercept)	490.08	464.91–515.25	**<0.001**	0.01	0.01–0.02	**<0.001**	128.49	98.65–158.33	**<0.001**
Presentation order	0.39	0.13–0.64	**<0.01**						
TSD condition at baseline (22:30)	−21.08	−55.49–13.33	0.23	0.46	0.24–0.89	**0.02**	−30.87	−72.04–10.31	0.14
Session 1 (08:00)	−1.31	−20.35–17.73	0.89	0.92	0.60–1.40	0.69	52.34	25.07–79.62	**<0.001**
Session 2 (12:30)	−12.13	−31.17–6.92	0.21	0.81	0.53–1.24	0.33	−15.68	−42.95–11.58	0.26
Session 3 (16:30)	10.67	−8.25–29.59	0.27	0.96	0.63–1.47	0.87	−19.30	−46.30–7.71	0.16
TSD	52.11	22.18–82.04	**<0.01**	2.28	1.51–3.45	**<0.001**	74.71	34.14–115.28	**<0.001**
TSD:Session 1 [TSD effect compared to session 3]							−87.67	−125.03 to −50.31	**<0.001**
TSD:Session 2 [TSD effect compared to session 3]							−39.53	−76.82 to −2.25	**0.04**
Observations	18,971						
Marginal *R*^2^	0.02						0.004

*Note: RT, response time in milliseconds; RTV, response time variation as measured by the standard deviation of the response time in milliseconds; CI, 95% confidence interval; TSD, total sleep deprivation; Marginal *R*^2^ represents the variance explained by the fixed-effects predictors (Nakagawa et al., 2017). A lapse was defined as responses taking at least 1,000 ms. Presentation order represents the order a given trial was within the test. All rows representing categorical variables report the effect compared to a reference level. The reference level for session was the baseline session (22:30) therefore all other effect estimates for different levels of session compare to performance at 22:30. The reference level for TSD was the normal sleep group. For models including an interaction between TSD and session, the effect estimate for TSD refers to the effect of TSD on performance at session 3. Interactions between TSD and session therefore refer to differences in the effect of TSD between the stated session (i.e., 1 or 2) and session 3*.

### Arithmetic Ability

**Figure 2:**
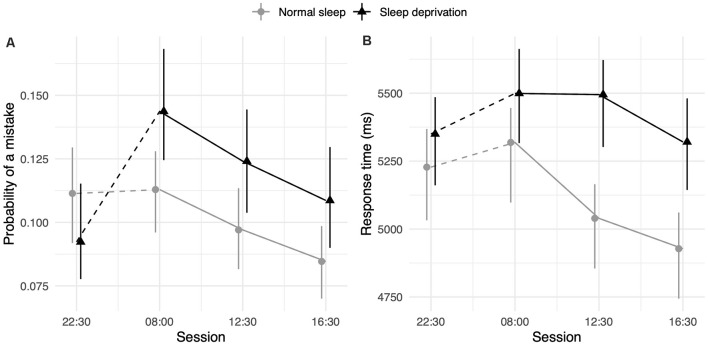
Arithmetic test performance depending on sleep condition and time-of-day. **(A)** Represents performance measured by the probability of making a mistake. **(B)** Represents performance measured by RT (in ms). Dots represent fixed-effect estimates and error bars represent bootstrapped confidence intervals with 100 iterations. The dotted line between 22:30 h and 08:00 h represents the night of total sleep deprivation for the sleep-deprivation group.

The sleep deprivation condition showed that a significant increase in odds of making mistakes during this test, as well as increased RTs. The odds of making a mistake on this test were significantly lower during the afternoon session (16:30 h) compared to baseline (22:30 h). The RTs were also significantly faster for the lunch (12:30 h) and afternoon (16:30 h) sessions compared to baseline (22:30 h). For RT, the effect of sleep deprivation was found to interact with time-of-day, such that the effect of sleep deprivation was smaller in the morning (08:00 h) compared to the afternoon (16:30 h). Full results for all outcomes can be seen in Table 2, and graphical representations of the effects can be found in Figure 2. The results of the model comparisons can be found in Supplementary Tables 5, 6.

**Table 2:** Arithmetic ability test performance predictions (odds ratios for making a mistake, and response times) based on sleep condition and time-of-day in the final mixed-effects model.

	Mistake			RT		
*Fixed-effect predictors*	*Odds ratios*	*CI*	*p*	*Estimates*	*CI*	*p*
(Intercept)	0.04	0.03–0.05	**<0.001**	5,610.75	5,392.14–5,829.35	**<0.001**
Presentation order	1.11	1.10–1.13	**<0.001**	−32.38	−39.15 to −25.61	**<0.001**
TSD condition at baseline (22:30)	0.81	0.58–1.13	0.21	122.00	−129.59–373.60	0.34
Session 1 (08:00)	1.02	0.81–1.28	0.90	90.74	−48.81–230.28	0.20
Session 2 (12:30)	0.86	0.68–1.08	0.18	−188.52	−323.67 to −53.36	**<0.01**
Session 3 (16:30)	0.74	0.59–0.93	**<0.01**	−300.16	−434.82 to −165.49	**<0.001**
TSD	1.32	1.04–1.68	**0.02**	392.82	150.26–635.38	**<0.01**
TSD:Session 1 [TSD effect compared to session 3]				−212.34	−398.36 to −26.32	**0.03**
TSD:Session 2 [TSD effect compared to session 3]				62.52	−118.46–243.51	0.50
Observations	11,085			
Marginal *R*^2^	0.15			0.03

*Note: RT, response time in milliseconds; CI, 95% confidence interval; TSD, total sleep deprivation; Marginal *R*^2^ represents the variance explained by the fixed-effects predictors (Nakagawa et al., 2017). Response time effects are in milliseconds (ms). Presentation order represents the order a given trial was within the test. All rows representing categorical variables report the effect compared to a reference level. The reference level for session was the baseline session (22:30 h) therefore all other effect estimates for different levels of session compare to performance at 22:30 h. The reference level for TSD was the normal sleep group. For models including an interaction between TSD and session, the effect estimate for TSD refers to the effect of TSD on performance at Session 3. Interactions between TSD and session therefore refer to differences in the effect of TSD between the stated Sessions (i.e., 1 or 2) and Session 3*.

### Episodic Memory

**Figure 3:**
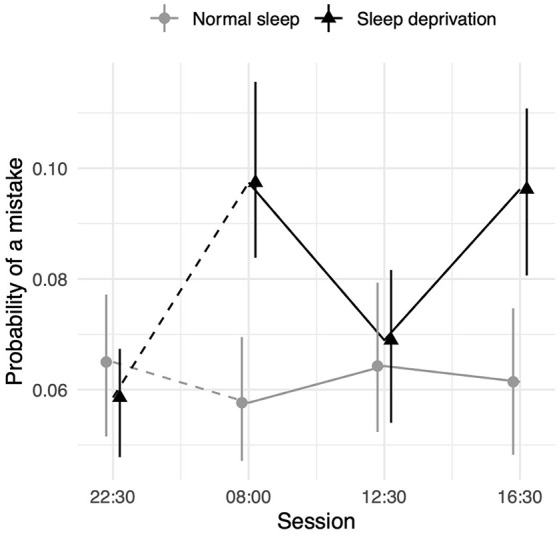
Episodic memory test performance as measured by the probability of making an misremembering any given word depending on sleep condition and time of day. Dots represent fixed-effect estimates and error bars represent bootstrapped confidence intervals with 100 iterations. The dotted line between 22:30 h and 08:00 h represents the night of total sleep deprivation for the sleep-deprivation group.

Sleep deprivation significantly increased the odds of misremembering a word. No significant time-of-day effect was observed. However, an interaction effect between the effect of sleep deprivation and time-of-day was observed. The effect of sleep deprivation was found to be smaller around lunch (12:30 h) relative to the afternoon (16:30 h). There was no significant difference between the effect of sleep deprivation in the morning (08:00 h) and that in the afternoon (16:30 h). A visualisation of these effects (see Figure 3) shows that performance was similar to the non-sleep deprived condition at this time. Full results for all outcomes can be seen in Table 3. The results of the model comparisons can be found in Supplementary Table 7.

**Table 3:** Episodic memory test performance (odds ratio for making a mistake) predictions based on sleep condition and time-of-day effects in the final mixed-effects model.

	Mistake		
*Fixed-effect predictors*	*Odds ratios*	*CI*	*p*
(Intercept)	0.21	0.16–0.27	**<0.001**
Within-test recall order [2 vs. 1]	0.47	0.44–0.52	**<0.001**
TSD condition at baseline (22:30)	0.90	0.67–1.20	0.46
Session 1 (08:00)	0.88	0.69–1.12	0.31
Session 2 (12:30)	0.99	0.78–1.25	0.92
Session 3 (16:30)	0.94	0.74–1.20	0.62
TSD	1.63	1.24–2.14	**<0.001**
TSD:Session 1 [TSD effect compared to session 3]	1.08	0.80–1.48	0.61
TSD:Session 2 [TSD effect compared to session 3]	0.66	0.49–0.90	**<0.01**
Observations	29,195
Marginal *R*^2^	0.05

*Note: CI, 95% confidence interval; TSD, total sleep deprivation; Marginal *R*^2^ represents the variance explained by the fixed-effects predictors (Nakagawa et al., 2017). Within-test recall order represents the effect of the second presentation of the words with new dummy words on performance. All rows representing categorical variables report the effect compared to a reference level. The reference level for session was the baseline session (22:30 h) therefore all other effect estimates for different levels of session compare to performance at 22:30 h. The reference level for TSD was the normal sleep group. For models including an interaction between TSD and session, the effect estimate for TSD refers to the effect of TSD on performance at Session 3. Interactions between TSD and session therefore refer to differences in the effect of TSD between the stated Session (i.e., 1 or 2) and Session 3*.

### Working Memory Test

**Figure 4:**
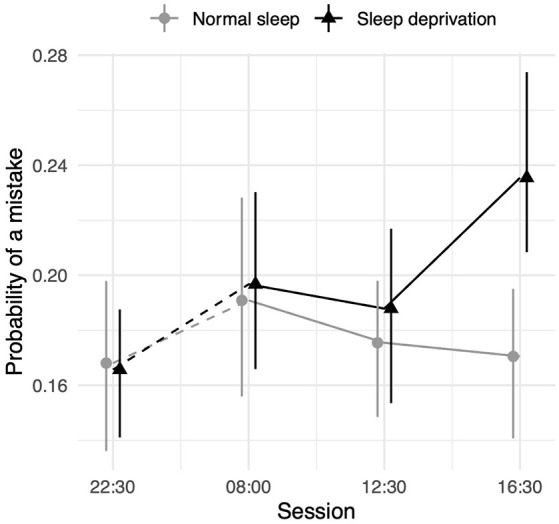
Working memory test performance as measured by the probability of making a mistake depending on sleep condition and time of day. Dots represent fixed-effect estimates and error bars represent bootstrapped confidence intervals with 100 iterations. The dotted line between 22:30 h and 08:00 h represents the night.

The sleep deprivation condition showed that a significant increase in the odds of making a working memory mistake. No significant time-of-day effect was observed for control participants. However, there was an interaction between sleep deprivation and time-of-day. The impact of sleep deprivation was found to be significantly lower in the morning session (08:00 h) compared to the afternoon session (16:30 h). The results regarding working memory can be found in Table 4, and visualisations of the predicted effects observed in the models can be found in Figure 4. The results of the model comparisons can be found in Supplementary Tables 8a,b.

**Table 4:** Working memory test performance predictions (odds ratio for making a mistake) based on sleep condition and time-of-day effects in the final mixed-effects model.

	Mistake		
*Fixed-effect predictors*	*Odds ratios*	*CI*	*p*
(Intercept)	0.16	0.13–0.21	**<0.001**
Presentation order	1.04	1.02–1.06	**<0.01**
TSD condition at baseline (22:30)	0.98	0.72–1.35	0.92
Session 1 (08:00)	1.17	0.88–1.54	0.28
Session 2 (12:30)	1.05	0.80–1.39	0.72
Session 3 (16:30)	1.02	0.77–1.34	0.90
TSD	1.50	1.13–1.99	**<0.01**
TSD:Session 1 [TSD effect compared to session 3]	0.69	0.49–0.99	**0.04**
TSD:Session 2 [TSD effect compared to session 3]	0.73	0.51–1.03	0.08
Observations	6,121
Marginal *R*^2^	0.01

*Note: CI, 95% confidence interval; TSD, total sleep deprivation; Marginal *R*^2^ represents the variance explained by the fixed-effects predictors (Nakagawa et al., 2017). Presentation order represents the order a given trial was within the test. All rows representing categorical variables report the effect compared to a reference level. The reference level for session was the baseline session (22:30 h) therefore all other effect estimates for different levels of session compare to performance at 22:30 h. The reference level for TSD was the normal sleep group. For models including an interaction between TSD and session, the effect estimate for TSD refers to the effect of TSD on performance at Session 3. Interactions between TSD and session therefore refer to differences in the effect of TSD between the stated Session (i.e., 1 or 2) and Session 3*.

### Stroop

**Figure 5:**
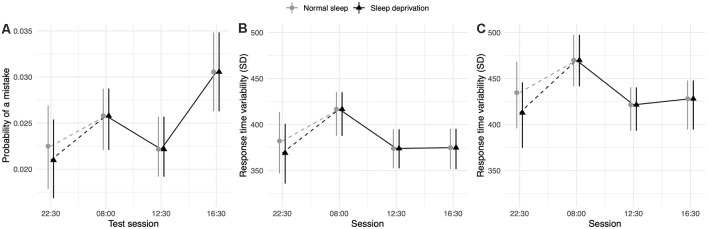
Strooptest performance depending on time-of-day. **(A)** Represents performance measured by the probability of making a mistake on the test. **(B)** Represents performance measured by the standard deviation of the difference in RT (in ms) between a cI and a preceding cC trial (cognitive conflict). **(C)** Represents performance measured by the standard deviation of the difference in RT (in ms) between aiI and a preceding cI trial (behavioural adjustment). Dots represent fixed-effect estimates and error bars represent bootstrapped confidence intervals with 100 iterations. The dotted line between 22:30 h and 08:00 h represents the night of total sleep deprivation for the sleep-deprivation group. Note that normal sleep and sleep deprivation conditions show identical estimates since the effect of sleep deprivation was not included in the model.

No effect of sleep deprivation was found for any measure of the Stroop test. The results of the model comparisons can be found in Supplementary Tables 9–13. Three base models, predicting mistakes, cognitive conflict RTV, and behavioural adjustment RTV, including time-of-day and sleep condition at baseline (22:30 h), outperformed intercept-only models. Participants were found to have higher odds of making errors at 16:30 h relative to performance at 22:30 h. No other significant time-of-day effects were observed. The results of these three models can be found in Table 5 and the model predictions of these three models are presented in Figure 5.

**Table 5:** Strooptest performance predictions based on sleep condition and time-of-day in the final mixed-effects models.

	Mistake			Cognitive conflict RTV			Behavioural adjustment RTV		
*Fixed-effect predictors*	*Odds ratios*	*CI*	*p*	*Estimates*	*CI*	*p*	*Estimates*	*CI*	*p*
(Intercept)	0.02	0.02–0.03	**<0.001**	382.12	348.41–415.84	**<0.001**	434.65	397.51–471.78	**<0.001**
TSD condition at baseline (22:30)	0.93	0.70–1.25	0.63	−13.01	−57.07–31.05	0.56	−21.90	−70.30–26.49	0.38
Session 1 (08:00)	1.15	0.90–1.47	0.26	34.51	−2.72–71.74	0.07	35.18	−5.56–75.92	0.09
Session 2 (12:30)	0.98	0.77–1.26	0.90	−8.07	−45.13–28.99	0.67	−13.21	−53.76–27.35	0.52
Session 3 (16:30)	1.37	1.08–1.74	**<0.01**	−7.21	−44.17–29.75	0.70	−6.63	−47.07–33.81	0.75
Observations	63,273						
Marginal *R*^2^	0.01			0.02			0.02

*Note: RTV, response time variation as measured by the standard deviation of the response time in milliseconds; CI, 95% confidence interval; TSD, total sleep deprivation; Marginal *R*^2^ represents the variance explained by the fixed-effects predictors (Nakagawa et al., 2017). All rows representing categorical variables report the effect compared to a reference level. The reference level for session was the baseline session (22:30 h) therefore all other effect estimates for different levels of session compare to performance at 22:30 h*.

### Sleepiness

**Figure 6:**
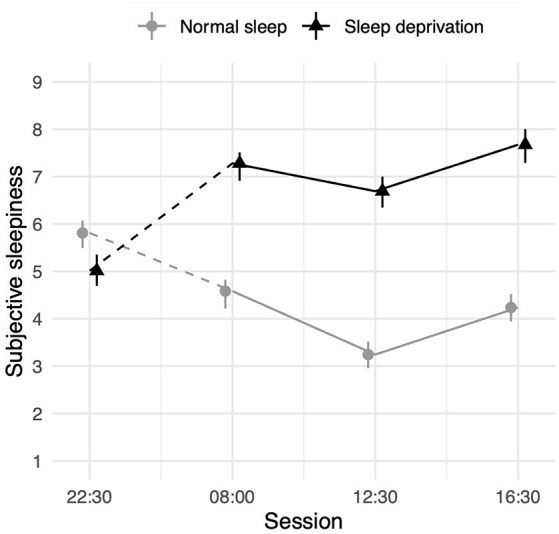
Subjective sleepiness (1 = very alert, 9 = very sleepy) depending on sleep condition and time- of-day. Dots represent fixed-effect estimates and error bars represent bootstrapped confidence intervals with 100 iterations. The dotted line between 22:30 h and 08:00 h represents the night of total sleep deprivation for the sleep-deprivation group.

Sleep deprivation was found to lead to a significant increase in subjective sleepiness. Sleepiness was also decreased compared to baseline (22:30 h) in all three daytime sessions. An interaction between sleep deprivation and time-of-day was also observed. The effect of sleep deprivation on subjective sleepiness was found to be significantly lower in the morning (08:00 h), compared to the afternoon (16:30 h). Full results for all outcomes can be seen in Table 6, and graphical representations of the effects can be found in Figure 6. The results of the model comparisons can be found in Supplementary Table 14.

**Table 6:** Subjective sleepiness score predictions based on sleep condition and time-of-day effects in the final mixed-effects model.

	Sleepiness rating		
*Fixed-effect predictors*	*Estimates*	*CI*	*p*
(Intercept)	5.81	5.47–6.16	**<0.001**
TSD condition at baseline (22:30)	−0.80	−1.27 to −0.33	**<0.01**
Session 1 (08:00)	−1.23	−1.63 to −0.83	**<0.001**
Session 2 (12:30)	−2.57	−2.97 to −2.17	**<0.001**
Session 3 (16:30)	−1.57	−1.97 to −1.17	**<0.001**
TSD	3.44	2.98–3.89	**<0.001**
TSD:Session 1 [TSD effect compared to session 3]	−0.74	−1.28 to −0.20	**<0.01**
TSD:Session 2 [TSD effect compared to session 3]	0.01	−0.53–0.55	0.97
Observations	
Marginal *R*^2^	0.46

*Note: CI, 95% confidence interval; TSD, total sleep deprivation; Marginal *R*^2^ represents the variance explained by the fixed-effects predictors (Nakagawa et al., 2017). All rows representing categorical variables report the effect compared to a reference level. The reference level for session was the baseline session (22:30 h) therefore all other effect estimates for different levels of session compare to performance at 22:30 h. The reference level for TSD was the normal sleep group. For models including an interaction between TSD and session, the effect estimate for TSD refers to the effect of TSD on performance at Session 3. Interactions between TSD and session therefore refer to differences in the effect of TSD between the stated Sessions (i.e., 1 or 2) and Session 3*.

### Cognitive Performance Before and Following Sleep Deprivation

**Figure 7:**
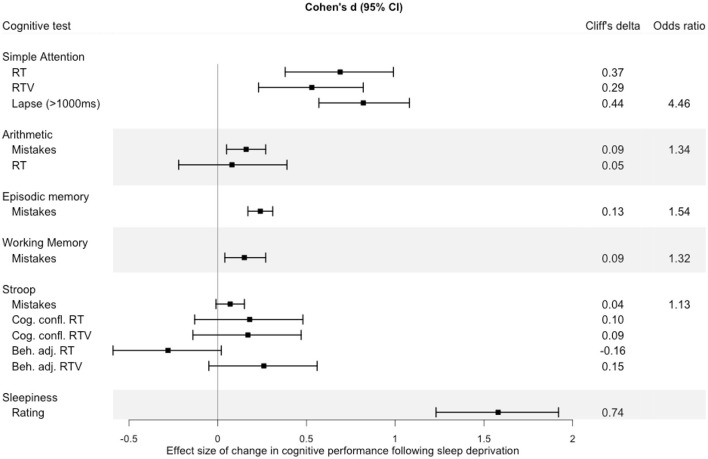
Effect size of change in performance from baseline (22:30 h) to performance after sleep deprivation (mean of performance at 08:00 h, 12:30 h, 16:30 h). Error bars represent 95% confidence intervals around the Cohen’s *d* value. The effect size estimates were calculated using the *compute.es* (Del Re, 2013) package in R, and plotted using the *forestplot* (Max and Lumley, 2020) package.

To illustrate the relative impact of sleep loss in the different cognitive domains measured in the KWA, effect sizes (Cohen’s *d*, Cliffs’s *d*, and odds ratio) were calculated on the change in average performance from before sleep deprivation (22:30 h) to after sleep deprivation (08:00 h, 12:30 h, 16:30 h). A forest plot (Figure 7) suggests that simple attention showed the largest effect size of the cognitive tests.

### Additional Analyses

At the suggestion of an anonymous reviewer, we further tested the effects of sleep deprivation on the simple attention task by running analyses predicting the count of lapses (rather than probability) as well as the count of false starts (pressing the button before a cue was given) as has been done previously (e.g., Grant et al., 2017). We observed that sleep deprivation significantly increased the number of lapses. There were not any time-of-day-related performance changes, nor an interaction between sleep deprivation and time-of-day (see Supplementary Tables 15, 16). We did not observe that sleep deprivation increased the number of false starts, though there was an increase in false starts during the final testing session (see Supplementary Tables 17, 18).

## Discussion

This study used the KWA, an ultra-short mobile cognitive testing battery, to assess the effect of sleep deprivation on cognitive performance at different times-of-day. One night of total sleep deprivation predicted significant performance impairments on four of the five cognitive tests measured, confirming the sensitivity of these 2-min tests to the effects of sleep loss. In other words, these data support that KWA can detect the cognitive effects of a 24-h increase in homeostatic sleep pressure. We additionally observed time-of-day effects on all cognitive tests measured, providing evidence of the sensitivity of 2-min tests to diurnal patterns in cognitive performance. These diurnal effects are likely driven by circadian processes, but also influenced by extrinsic influences such as food intake and light levels, as well as the internal influence of increasing time awake; when performing the KWA in the afternoon, all participants had been awake longer than when performing the KWA in the morning.

Following our expectations, sleep deprivation led to a clear impairment in simple attention response time. Despite the brevity of the test, we also showed that sleep-deprived participants showed the expected increase in lapses as well as greater RTV, results that match the predictions of the state-instability hypothesis (Doran et al., 2001). These results are similar to previous attempts to make touchscreen attention tasks (Grant et al., 2017; Arsintescu et al., 2019). One key difference is that we did not observe an increase in false starts as had been shown in a previous validation of a touchscreen version of the PVT (Grant et al., 2017) which may be due the relative rarity of these events, the even shorter duration of our task (the KWA being 2-min vs. 3-min in the cited study), as well as differences in study design. The significant impact of sleep deprivation on RTV suggests that enough data is collected per 2-min trial not only to detect the increase in intra-individual variability in ability to sustain attention following sleep deprivation but also detect that the sleep deprivation effect was time-of-day dependent. This highlights the potential of measuring intra-individual performance attention performance using ultra-brief tasks, which may increase accuracy if used to predicting sleepiness or other altered cognitive states and phenotypes (Frey et al., 2004; Vaurio et al., 2009; Hill et al., 2013). This is especially important for understanding how sleep loss affects different psychiatric populations. For example, it has been shown that ADHD is associated with a delayed circadian rhythm and sleep problems (Van Veen et al., 2010; Coogan and McGowan, 2017) and that sleep loss affects cognition in subjects with high ADHD-symptoms more than others (Gruber et al., 2011; Moreau et al., 2013; Floros et al., 2021; Holingue et al., 2021). Also, sleep loss may induce mania in patients with bipolar disorder (Stevens et al., 2014) and psychotic symptoms (Waters et al., 2018). However, we know very little about how this negative effect evolves during the day.

Sleep deprivation caused poorer performance on the arithmetic test, in line with evidence shown from similar tasks following sleep deprivation (Carskadon and Dement, 1979; Frey et al., 2004). Arithmetic accuracy and RT improved over time in the well-rested group, suggesting learning effects, or alternatively a circadian-driven improvement in arithmetic performance throughout the day. However, these improvements overtime were less observable in sleep-deprived participants. The evidence of a positive time effects in the well-rested condition makes it difficult to interpret whether sleep deprivation is affecting performance on the test, or instead is interfering with learning the test. Arecent study found a similar improvement in an addition task over testing days, however not when participants were experience circadian misalignment (simulating shift work) potentially supporting a theorisation that sleep loss or circadian misalignment is impairing learning effects (Chellappa et al., 2018). Despite our participants having practiced the tests twice prior to the test-day, future studies may benefit from participants practicing more times to reduce the impact of learning effects.

Again, following our expectation, sleep deprivation led to a decrease in episodic memory ability. Time-of-day showed an effect particularly in the sleep-deprived group, where participants showed a peak in performance around midday. Performance at this point became nearly in distinguishable from the well-rested group. However, performance quickly deteriorated again in the afternoon. Previous evidence of the impact of sleep loss on word-list episodic memory tasks has been mixed, and one reason suggested that this is the “task impurity problem” (Miyake et al., 2000; de Almeida Valverde Zanini et al., 2012), whereby cognitive tasks often rely on multiple cognitive faculties. Specifically relating to word-list episodic memory tasks, there may be the influence on both short- and long-term memory relating to when the word was presented. While testing multiple cognitive components may predict real-life performance better than testing only single components (Vestberg et al., 2017), using tests that involve many simultaneous cognitive components complicate the process of teasing out which component(s) that is affected by a particular manipulation. In our task all words were presented simultaneously, and a control variable was added to the model to account for the two presentation trials, which may reduce the influence of different forms of memory being used. Nonetheless, the task impurity problem represents a consistent problem which should be taken into account when interpreting performance impairments in cognitive tasks. A further reason for mixed evidence may be due to strong time-of-day effects on episodic memory performance in sleep deprived individuals. Indeed, it is interesting that the KWA was able to observe that time-of-day appears to be particularly influential for sleep-deprived participants, while well-rested participants showed no noticeable time-of-day effects. While we aimed to analyse these interaction effects in an exploratory manner, the results are nonetheless consistent with previous research (Lo et al., 2012) showing circadian modulation of the sleep loss effect on cognition. More broadly, these results suggest that interaction effects between time awake and diurnal influences can be measured using ultra-short tests.

Sleep deprivation caused impairments in working-memory capacity. This effect seems to be driven by a particularly large decrease in the afternoon, while the impairments seen in the morning and at midday were more modest. Similar to the interaction effects found in the episodic memory test, the stronger time of day effects in sleep deprived may provide an explanation as to why there has been inconsistent findings regarding the effect of sleep deprivation on visual working memory (Drummond et al., 2012).

Finally, sleep deprivation was not shown to impact Stroop performance. While we expected Stroop performance to be impaired, we also expected the effect size of sleep deprivation on Stroop performance to be among the smallest of the cognitive tests. Our results potentially conflict with some published evidence showing significant detrimental effects of sleep loss focusing on top-down adaptation (Gevers et al., 2015) as well as within specific subpopulations (Labelle et al., 2015; Floros et al., 2021). However, the results of this study are corroborated by a number of previous studies that also do not find such an effect (Sagaspe et al., 2006; Cain et al., 2011; Bratzke et al., 2012; Patrick et al., 2017). One reason we did not find similar significant effects as previous studies is due to differences in the way of analysing the data (in the case of Gevers et al., 2015) or our use of a young healthy sample. However, a longer version of the Stroop task involving faces showed that an effect of sleep deprivation using similar measurements (cognitive conflict RTV) in the same study population (Floros et al., 2021). Therefore, an alternative reason for not finding an effect could relate to differences in the duration of the Stroop task, where longer tests include more data points, and thus better power to detect smaller effects, as well as an increased task-induced fatigue. Additionally, while participants had a practice session where they discussed possible problems carrying out the tests with a research assistant, it is possible that colour-blindness in participants was missed, hence increasing variability.

There are a number of limitations to our procedure which provide avenues for future research. In the present study, participants only completed the cognitive tests at three points following sleep or sleep deprivation. While we were able to show significant time-of-day patterns in cognitive performance for some tests, future studies wishing to assess time-of-day trajectories in cognitive functioning would benefit from more measurements per day. For example, administration of the KWA later into the evening would also allow for measurement of performance during the wake maintenance zone, where task-specific preservation effects on cognitive performance following sleep loss has been shown (McMahon et al., 2018). Though researchers should also consider that this may lead to an increase in practice effects, which in turn may be differentially impacted following sleep loss. A weakness of the study design is that performance at the three daytime sessions was compared to the previous night at 22:30 h, a time when cognitive performance is normally not at its best. Therefore, while we refer to this as a “baseline,” it does not represent peak cognitive performance. Although this has been taken into account in the interpretation of the results, future studies could avoid this problem by collecting more datapoints across the day.

While the aim of this study was to describe sleep-related and diurnal changes in KWA performance, it is important to note that we did not measure or strictly control for exogenous factors, such as physical activity, intake of food, and light exposure. We provided similar meals (lunch and afternoon snack) and kept participants in a constantly lit sleep lab for the majority of the day to reduce exogenous influences. Nonetheless, the time-of-day effects are likely a result of both circadian processes and differences in behaviour. We also observed slight differences in the timing of the tests between conditions. Most of these differences were very small, however, the morning session differed by an average of 48 min between conditions, with sleep-deprived participants tending to complete the KWA earlier. An additional problem with the morning session, is that testing occurred on average at 08:06 h for the control group, while these participants reported waking up on average at 07:37 h (according to actigraphy measurements) which may have resulted in some residual sleep inertia which reduced cognitive performance. These issues perhaps characterise one of the downsides of ambulatory cognitive measurement, where procedure standardisation is less enforceable than in traditional lab conditions. However, with the possibility of collecting more data using less resources, such downsides may be offset by increase in statistical power provided by larger datasets. A related limitation is that we allowed participants to use their own smartphone. While there is no reason to believe that this would have a systematic effect on our key outcomes in this study due to individuals being randomised into respective sleep conditions, it may increase inter-individual response variance since some older smartphones have slower touchscreen reaction speed latencies. On the other hand, this made it possible to participants to practice at home, and to be comfortable using the device. Nonetheless, if the KWA should be used to specifically investigate inter-individual differences in cognitive performance in future, it is recommended that all participants use the same model smartphone.

This study has a number of implications for both cognitive testing and daily performance in general. First, we show that tests as short as 2 min can be used to effectively measure the effects of sleep deprivation on a number of core cognitive functions. Using mobile devices (e.g., smartphones) for these brief tests provide a means for measuring cognition throughout the day and in unexplored environments, as well as decreasing the likelihood of test-induced fatigue. Such tests would be particularly useful in field studies within the medical, transport, or military disciplines, where brief time demands as well as minimal equipment requirements provide obvious benefits. The findings also have relevance for the scheduling of work tasks in order to keep performance as high as possible. For example, for a sleep-deprived person, a period around midday may be the best time to schedule tasks requiring episodic memory whereas for someone well-rested, performance is likely to be stable throughout the day. An ultra-short cognitive testing battery such as the KWA could potentially also be used to investigate inter-individual cognitive traits, which have been shown to be useful in predicting vulnerability to sleep deprivation (Van Dongen et al., 2012; Patanaik et al., 2015). Finally, these tests may be used in clinical assessments of neuropsychiatric disorders such as attention deficit hyperactivity disorder (ADHD), where cognitive capacity may be dependent both on sleep length and quality as well as other contextual factors including stress (Floros et al., 2021).

To conclude, a brief battery of 2-min smartphone-based tests was able to detect significant impairments on four out of five cognitive tests measured following sleep deprivation. The effects of sleep deprivation appear to show dependency on time of day, often differing from the time-of-day effects seen in well-rested controls. The results have several implications—from highlighting the possibilities of cognitive testing using smartphones, to the potential benefit of scheduling tasks in order to best preserve cognitive ability following sleep deprivation.

## Data Availability Statement

The datasets presented in this study can be found in online repositories. The names of the repository/repositories and accession number(s) can be found below: https://doi.org/10.5281/zenodo.4081465.

## Ethics Statement

The studies involving human participants were reviewed and approved by Stockholm Regional Ethical Review Board. The patients/participants provided their written informed consent to participate in this study.

## Author Contributions

BH wrote the article in consultation with MI, PP, TS and JA. JA conceived and designed the cognitive testing application. TS and JA conceived and designed the sleep deprivation experiment. BH, TS and JA collected the data. BH analysed the data, in consultation with MI. All authors contributed to the article and approved the submitted version.

## Conflict of Interest

The authors declare that the research was conducted in the absence of any commercial or financial relationships that could be construed as a potential conflict of interest.

## Footnotes

^1^The PVT requires participants to respond to visual stimuli presented at random intervals and records the response times. The standard PVT lasts for 10 min. It is commonly referred to as the “gold standard” of behavioural alertness measurements.
