# Supplementary material for: Quantifying Cognitive Impairment After Sleep Deprivation at Different Times of Day: A Proof of Concept Using Ultra-Short Smartphone-Based Tests
Source: Front Behav Neurosci. 2021 Apr 13;15:666146. doi: 10.3389/fnbeh.2021.666146 (PMC8076531; doi:10.3389/fnbeh.2021.666146)
Supplement: Supplementary file 1 [file Table_1.DOCX]

**SUPPLEMENTAL INFORMATION**

**Table S1. Descriptive statistics of test timing (in hours and minutes) between conditions**

| *Session* | *Well-restred control* | | *Sleep deprivation* | |
| --- | --- | --- | --- | --- |
|  | Mean | Standard deviation | Mean | Standard deviation |
| Baseline | 22:43 | 0:50 | 22:34 | 0:23 |
| 1 | 08:06 | 0:54 | 07:18 | 0:37 |
| 2 | 12:34 | 0:10 | 12:34 | 0.05 |
| 3 | 16:37 | 0:22 | 16:39 | 0:23 |

| **Table S2. Simple attention reaction-time model comparisons** | | | | | | | | |
| --- | --- | --- | --- | --- | --- | --- | --- | --- |
|  | *DF* | *AIC* | *BIC* | *Log-Likelihood* | *Deviance* | *Chi-square* | *Chi DF* | *p* |
| Intercept-only | 4 | 246629.78 | 246661.19 | -123310.89 | 246621.78 |  |  |  |
| Base | 9 | 246557.09 | 246627.75 | -123269.55 | 246539.09 | 82.69 | 5 | <.001 |
| **Sleep Deprivation** | **10** | **246547.78** | **246626.29** | **-123263.89** | **246527.78** | **11.31** | **1** | **<.001** |
| Sleep Deprivation x Session | 12 | 246550.87 | 246645.08 | -123263.44 | 246526.87 | 0.91 | 2 | .63 |
| Note. Models are compared against the previous level of model complexity. A base model represents a model with condition at baseline (22:00) and other factors, such as session (time-of-day) and order of stimulus within a given test. Interaction represents an interaction between the effect of sleep deprivation and session (time-of-day). | | | | | | | | |

| **Table S3. Simple attention lapses model comparisons** | | | | | | | | |
| --- | --- | --- | --- | --- | --- | --- | --- | --- |
|  | *DF* | *AIC* | *BIC* | *Log-Likelihood* | *Deviance* | *Chi-square* | *Chi DF* | *p* |
| Intercept-only | 3 | 3451.17 | 3474.71 | -1722.59 | 3445.17 |  |  |  |
| Base | 7 | 3438.24 | 3493.15 | -1712.12 | 3424.24 | 20.93 | 4 | <.001 |
| **Sleep Deprivation** | **8** | **3424.76** | **3487.51** | **-1704.38** | **3408.76** | **15.49** | **1** | **<.001** |
| Sleep Deprivation x Session | 10 | 3428.37 | 3506.82 | -1704.19 | 3408.37 | 0.38 | 2 | .83 |
| Note. Models are compared against the previous level of model complexity. A base model represents a model with condition at baseline (22:00) and other factors, such as session (time-of-day) and order of stimulus within a given test. Interaction represents an interaction between the effect of sleep deprivation and session (time-of-day). | | | | | | | | |

| **Table S4. Simple attention reaction-time variation model comparisons** | | | | | | | | |
| --- | --- | --- | --- | --- | --- | --- | --- | --- |
|  | *DF* | *AIC* | *BIC* | *Log-Likelihood* | *Deviance* | *Chi-square* | *Chi DF* | *p* |
| Intercept-only | 3 | 287164.31 | 287187.86 | -143579.15 | 287158.31 |  |  |  |
| Base | 7 | 287113.05 | 287168.01 | -143549.53 | 287099.05 | 59.26 | 4 | <.001 |
| Sleep Deprivation | 8 | 287111.59 | 287174.39 | -143547.79 | 287095.59 | 3.46 | 1 | .06 |
| **Sleep Deprivation x Session** | **10** | **287094.41** | **287172.91** | **-143537.2** | **287074.41** | **21.18** | **2** | **<.001** |
| Note. Models are compared against the previous level of model complexity. A base model represents a model with condition at baseline (22:00) and other factors, such as session (time-of-day) and order of stimulus within a given test. Interaction represents an interaction between the effect of sleep deprivation and session (time-of-day). | | | | | | | | |

| **Table S5. Arithmetic accuracy model comparisons** | | | | | | | | |
| --- | --- | --- | --- | --- | --- | --- | --- | --- |
|  | *DF* | *AIC* | *BIC* | *Log-Likelihood* | *Deviance* | *Chi-square* | *Chi DF* | *p* |
| Intercept-only | 4 | 7767.62 | 7796.87 | -3879.81 | 7759.62 |  |  |  |
| Base | 9 | 7575.23 | 7641.05 | -3778.62 | 7557.23 | 202.39 | 5 | <.001 |
| **Sleep Deprivation** | **10** | **7572.23** | **7645.37** | **-3776.12** | **7552.23** | **5** | **1** | **.025** |
| Sleep Deprivation x Session | 12 | 7574 | 7661.76 | -3775 | 7550 | 2.23 | 2 | .33 |
| Note. Models are compared against the previous level of model complexity. A base model represents a model with condition at baseline (22:00) and other factors, such as session (time-of-day) and order of stimulus within a given test. Interaction represents an interaction between the effect of sleep deprivation and session (time-of-day). | | | | | | | | |

| **Table S6. Arithmetic reaction-time model comparisons** | | | | | | | | |
| --- | --- | --- | --- | --- | --- | --- | --- | --- |
|  | *DF* | *AIC* | *BIC* | *Log-Likelihood* | *Deviance* | *Chi-square* | *Chi DF* | *p* |
| Intercept-only | 5 | 198302.54 | 198339.12 | -99146.27 | 198292.54 |  |  |  |
| Base | 10 | 198193.7 | 198266.86 | -99086.85 | 198173.7 | 118.84 | 5 | <.001 |
| Sleep Deprivation | 11 | 198186.33 | 198266.8 | -99082.16 | 198164.33 | 9.37 | 1 | <.01 |
| **Sleep Deprivation x Session** | **13** | **198181.36** | **198276.47** | **-99077.68** | **198155.36** | **8.96** | **2** | **.011** |
| Note. Models are compared against the previous level of model complexity. A base model represents a model with condition at baseline (22:00) and other factors, such as session (time-of-day) and order of stimulus within a given test. Interaction represents an interaction between the effect of sleep deprivation and session (time-of-day). | | | | | | | | |

| **Table S7. Short-term memory accuracy model comparisons** | | | | | | | | |
| --- | --- | --- | --- | --- | --- | --- | --- | --- |
|  | *DF* | *AIC* | *BIC* | *Log-Likelihood* | *Deviance* | *Chi-square* | *Chi DF* | *p* |
| Intercept-only | 4 | 17117.12 | 17150.25 | -8554.56 | 17109.12 |  |  |  |
| Base | 9 | 16801.49 | 16876.03 | -8391.75 | 16783.49 | 325.63 | 5 | <.001 |
| Sleep Deprivation | 10 | 16791.58 | 16874.4 | -8385.79 | 16771.58 | 11.92 | 1 | <.001 |
| **Sleep Deprivation x Session** | **12** | **16784.98** | **16884.36** | **-8380.49** | **16760.98** | **10.6** | **2** | **<.01** |
| Note. Models are compared against the previous level of model complexity. A base model represents a model with condition at baseline (22:00) and other factors, such as session (time-of-day) and order of stimulus within a given test. Interaction represents an interaction between the effect of sleep deprivation and session (time-of-day). | | | | | | | | |

| **Table S8a. Working memory accuracy model comparisons** | | | | | | | | |
| --- | --- | --- | --- | --- | --- | --- | --- | --- |
|  | *DF* | *AIC* | *BIC* | *Log-Likelihood* | *Deviance* | *Chi-square* | *Chi DF* | *p* |
| Intercept-only | 2 | 6012.59 | 6026.03 | -3004.29 | 6008.59 |  |  |  |
| **Base** | **7** | **6003.11** | **6050.15** | **-2994.56** | **5989.11** | **19.48** | **5** | **<.01** |
| Sleep Deprivation | 8 | 6002.05 | 6055.81 | -2993.02 | 5986.05 | 3.06 | 1 | .08 |
| Sleep Deprivation x Session | 10 | 6001.11 | 6068.31 | -2990.56 | 5981.11 | 4.94 | 2 | .09 |
| Note. Models are compared against the previous level of model complexity. A base model represents a model with condition at baseline (22:00) and other factors, such as session (time-of-day) and order of stimulus within a given test. Interaction represents an interaction between the effect of sleep deprivation and session (time-of-day). | | | | | | | | |

| **Table S8b. Working memory accuracy model comparisons** | | | | | | | | |
| --- | --- | --- | --- | --- | --- | --- | --- | --- |
|  | *DF* | *AIC* | *BIC* | *Log-Likelihood* | *Deviance* | *Chi-square* | *Chi DF* | *p* |
| Base | 7 | 6003.11 | 6050.15 | -2994.56 | 5989.11 |  |  |  |
| **Sleep Deprivation x Session** | **10** | **6001.11** | **6068.31** | **-2990.56** | **5981.11** | **8** | **3** | **.05** |
| Note. Models are compared against the previous level of model complexity. A base model represents a model with condition at baseline (22:00) and other factors, such as session (time-of-day) and order of stimulus within a given test. Interaction represents an interaction between the effect of sleep deprivation and session (time-of-day). | | | | | | | | |

| **Table S9. Stroop mistakes model comparisons** | | | | | | | | |
| --- | --- | --- | --- | --- | --- | --- | --- | --- |
|  | *DF* | *AIC* | *BIC* | *Log-Likelihood* | *Deviance* | *Chi-square* | *Chi DF* | *p* |
| Intercept-only | 3 | 16922.8 | 16949.97 | -8458.4 | 16916.8 |  |  |  |
| **Base** | **7** | **16911.19** | **16974.58** | **-8448.6** | **16897.19** | **19.61** | **4** | **<.001** |
| Sleep Deprivation | 8 | 16910.34 | 16982.79 | -8447.17 | 16894.34 | 2.85 | 1 | .09 |
| Sleep Deprivation x Session | 10 | 16912.75 | 17003.3 | -8446.37 | 16892.75 | 1.6 | 2 | .45 |
| Note. Models are compared against the previous level of model complexity. A base model represents a model with condition at baseline (22:00) and other factors, such as session (time-of-day) and order of stimulus within a given test. Interaction represents an interaction between the effect of sleep deprivation and session (time-of-day). | | | | | | | | |

| **Table S10. Stroop cognitive conflict reaction-time model comparisons** | | | | | | | | |
| --- | --- | --- | --- | --- | --- | --- | --- | --- |
|  | *DF* | *AIC* | *BIC* | *Log-Likelihood* | *Deviance* | *Chi-square* | *Chi DF* | *p* |
| **Intercept-only** | **3** | **167434.09** | **167456.09** | **-83714.05** | **167428.09** |  |  |  |
| Base | 7 | 167438.64 | 167489.97 | -83712.32 | 167424.64 | 3.45 | 4 | .49 |
| Sleep Deprivation | 8 | 167439.86 | 167498.52 | -83711.93 | 167423.86 | 0.78 | 1 | .38 |
| Sleep Deprivation x Session | 10 | 167440.65 | 167513.97 | -83710.33 | 167420.65 | 3.21 | 2 | .20 |
| Note. Models are compared against the previous level of model complexity. A base model represents a model with condition at baseline (22:00) and other factors, such as session (time-of-day) and order of stimulus within a given test. Interaction represents an interaction between the effect of sleep deprivation and session (time-of-day). | | | | | | | | |

| **Table S11. Stroop cognitive conflict reaction-time variation model comparisons** | | | | | | | | |
| --- | --- | --- | --- | --- | --- | --- | --- | --- |
|  | *DF* | *AIC* | *BIC* | *Log-Likelihood* | *Deviance* | *Chi-square* | *Chi DF* | *p* |
| Intercept-only | 3 | 8003.42 | 8016.75 | -3998.71 | 7997.42 |  |  |  |
| **Base** | **7** | **7998.23** | **8029.31** | **-3992.11** | **7984.23** | **13.2** | **4** | **.01** |
| Sleep Deprivation | 8 | 7998.87 | 8034.4 | -3991.44 | 7982.87 | 1.36 | 1 | .24 |
| Sleep Deprivation x Session | 10 | 8001.77 | 8046.17 | -3990.88 | 7981.77 | 1.11 | 2 | .58 |
| Note. Models are compared against the previous level of model complexity. A base model represents a model with condition at baseline (22:00) and other factors, such as session (time-of-day) and order of stimulus within a given test. Interaction represents an interaction between the effect of sleep deprivation and session (time-of-day). | | | | | | | | |

| **Table S12. Stroop behavioural adjustment reaction-time model comparisons** | | | | | | | | |
| --- | --- | --- | --- | --- | --- | --- | --- | --- |
|  | *DF* | *AIC* | *BIC* | *Log-Likelihood* | *Deviance* | *Chi-square* | *Chi DF* | *p* |
| **Intercept-only** | **3** | **128064.41** | **128085.56** | **-64029.21** | **128058.41** |  |  |  |
| Base | 7 | 128068.32 | 128117.66 | -64027.16 | 128054.32 | 4.09 | 4 | .39 |
| Sleep Deprivation | 8 | 128069.44 | 128125.83 | -64026.72 | 128053.44 | 0.88 | 1 | .35 |
| Sleep Deprivation x Session | 10 | 128072.72 | 128143.21 | -64026.36 | 128052.72 | 0.72 | 2 | .70 |
| Note. Models are compared against the previous level of model complexity. A base model represents a model with condition at baseline (22:00) and other factors, such as session (time-of-day) and order of stimulus within a given test. Interaction represents an interaction between the effect of sleep deprivation and session (time-of-day). | | | | | | | | |

| **Table S13. Stroop behavioural adjustment reaction-time variation model comparisons** | | | | | | | | |
| --- | --- | --- | --- | --- | --- | --- | --- | --- |
|  | *DF* | *AIC* | *BIC* | *Log-Likelihood* | *Deviance* | *Chi-square* | *Chi DF* | *p* |
| Intercept-only | 3 | 8145.64 | 8158.97 | -4069.82 | 8139.64 |  |  |  |
| **Base** | **7** | **8140.05** | **8171.15** | **-4063.02** | **8126.05** | **13.59** | **4** | **<.01** |
| Sleep Deprivation | 8 | 8138.85 | 8174.39 | -4061.42 | 8122.85 | 3.2 | 1 | .07 |
| Sleep Deprivation x Session | 10 | 8141.3 | 8185.73 | -4060.65 | 8121.3 | 1.54 | 2 | .46 |
| Note. Models are compared against the previous level of model complexity. A base model represents a model with condition at baseline (22:00) and other factors, such as session (time-of-day) and order of stimulus within a given test. Interaction represents an interaction between the effect of sleep deprivation and session (time-of-day). | | | | | | | | |

| **Table S14. KSS score model comparisons** | | | | | | | | |
| --- | --- | --- | --- | --- | --- | --- | --- | --- |
|  | *DF* | *AIC* | *BIC* | *Log-Likelihood* | *Deviance* | *Chi-square* | *Chi DF* | *p* |
| Intercept-only | 4 | 8674.61 | 8698.8 | -4333.3 | 8666.61 | NA |  |  |
| Base | 8 | 8483.06 | 8531.44 | -4233.53 | 8467.06 | 199.55 | 4 | <0.001 |
| Sleep Deprivation | 9 | 8287.73 | 8342.17 | -4134.86 | 8269.73 | 197.33 | 1 | <0.001 |
| **Sleep Deprivation x Session** | **11** | **8282.26** | **8348.79** | **-4130.13** | **8260.26** | **9.47** | **2** | **<.01** |
| Note. Models are compared against the previous level of model complexity. A base model represents a model with condition at baseline (22:00) and other factors, such as session (time-of-day) and order of stimulus within a given test. Interaction represents an interaction between the effect of sleep deprivation and session (time-of-day). | | | | | | | | |

| **Table S15. Simple Attention Lapses (Counts) Model Comparions** | | | | | | | | |
| --- | --- | --- | --- | --- | --- | --- | --- | --- |
|  | *DF* | *AIC* | *BIC* | *Log.Likelihood* | *Deviance* | *Chi.square* | *Chi.DF* | *p.value* |
| Intercept-only | NA | 2297.51 | 2311.07 | -1145.76 | 2291.51 | NA | NA | NA |
| Base | 4 | 2290.13 | 2321.76 | -1138.06 | 2276.13 | 15.39 | 4 | 0.004 |
| **Sleep Deprivation** | **1** | **2279.89** | **2316.04** | **-1131.95** | **2263.89** | **12.23** | **1** | **<0.001** |
| Sleep Deprivation x Session | 2 | 2283.51 | 2328.71 | -1131.76 | 2263.51 | 0.38 | 2 | 0.829 |
| *Models are compared against the previous level of model complexity. A base model repesents a model with condition at baseline (22:00) and other factors, such as session (time-of-day) and order of stimulus within a given test. Interaction represents a interaction between the effect of sleep deprivation and time-of-day (session)* | | | | | | | | |

| **Table S16. Simple Attention Lapses (Counts)** | | | |
| --- | --- | --- | --- |
|  |  | | |
| *Predictors* | *Estimates* | *CI* | *p* |
| (Intercept) | 0.45 | 0.16 – 0.74 | **0.003** |
| TSD condition at baseline (22:30) | -0.20 | -0.60 – 0.20 | 0.329 |
| Session 1 (08:00) | -0.02 | -0.35 – 0.31 | 0.899 |
| Session 2 (12:30) | -0.07 | -0.41 – 0.26 | 0.663 |
| Session 3 (16:30) | 0.01 | -0.32 – 0.34 | 0.941 |
| TSD | 0.50 | 0.22 – 0.77 | **<0.001** |
| N _id_ | 177 | | |
| Observations | 678 | | |
| Marginal R^2^ / Conditional R^2^ | 0.039 / 0.254 | | |

| **Table S17. Simple Attention False Starts (Counts) Model Comparions** | | | | | | | | |
| --- | --- | --- | --- | --- | --- | --- | --- | --- |
|  | *DF* | *AIC* | *BIC* | *Log.Likelihood* | *Deviance* | *Chi.square* | *Chi.DF* | *p.value* |
| Intercept-only | NA | 2061.42 | 2074.98 | -1027.71 | 2055.42 | NA | NA | NA |
| **Base** | **4** | **2044.72** | **2076.35** | **-1015.36** | **2030.72** | **24.70** | **4** | **<0.001** |
| Sleep Deprivation | 1 | 2044.04 | 2080.19 | -1014.02 | 2028.04 | 2.68 | 1 | 0.10 |
| Sleep Deprivation x Session | 2 | 2047.60 | 2092.79 | -1013.80 | 2027.60 | 0.43 | 2 | 0.80 |
| *Models are compared against the previous level of model complexity. A base model repesents a model with condition at baseline (22:00) and other factors, such as session (time-of-day) and order of stimulus within a given test. Interaction represents a interaction between the effect of sleep deprivation and time-of-day (session)* | | | | | | | | |

| **Table S18. Simple Attention False Starts (Counts)** | | | |
| --- | --- | --- | --- |
|  |  | | |
| *Predictors* | *Estimates* | *CI* | *p* |
| (Intercept) | 0.74 | 0.49 – 0.98 | **<0.001** |
| TSD condition at baseline (22:30) | -0.24 | -0.55 – 0.07 | 0.126 |
| Session 1 (08:00) | 0.05 | -0.21 – 0.30 | 0.728 |
| Session 2 (12:30) | -0.08 | -0.33 – 0.18 | 0.556 |
| Session 3 (16:30) | 0.30 | 0.05 – 0.55 | **0.020** |
| N _id_ | 177 | | |
| Observations | 678 | | |
| Marginal R^2^ / Conditional R^2^ | 0.021 / 0.450 | | |


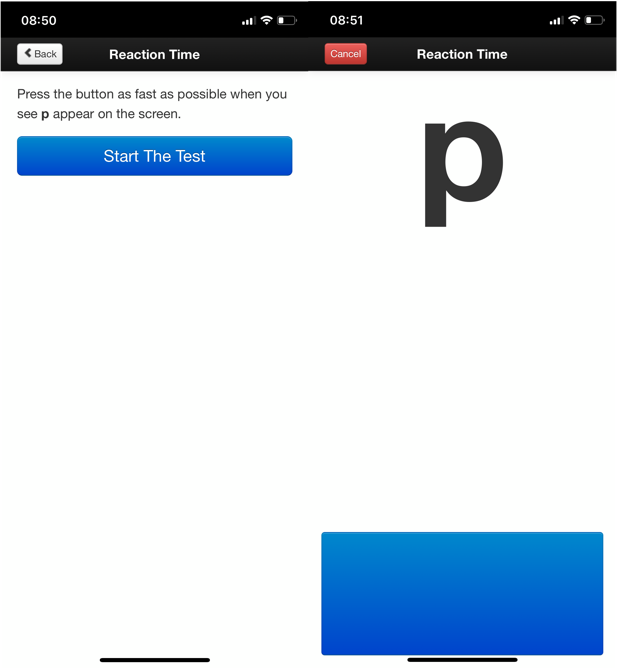


Figure S1. Images taken from the simple attention test. Left shows the instructions given to participant. Right shows an example of ‘p’ stimuli and blue box where participants should click as fast as possible.


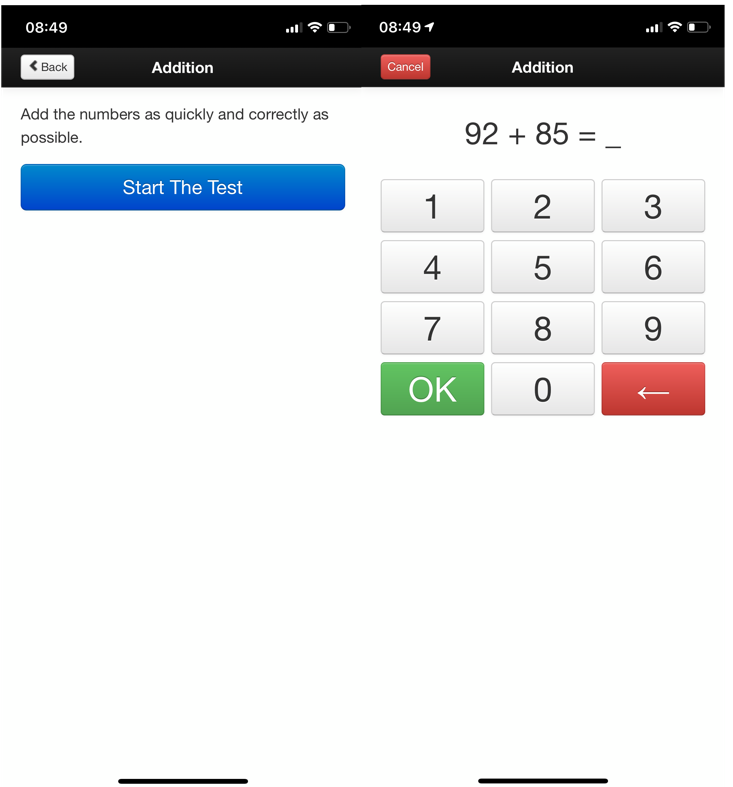


Figure S2. Images taken from the arithmetic test. Left shows the instructions given to participant. Right shows an example of a addition question and buttons where participant should provide the answer.


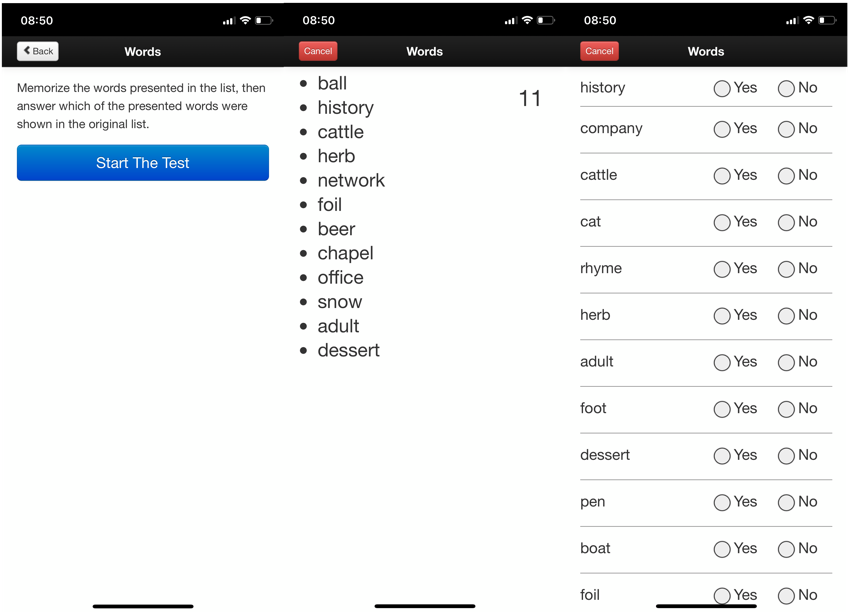


Figure S3. Images taken from the episodic memory test. Left shows the instructions given to participant. Middle shows an example of the list of words that participants are asked to memorise. Right shows an example of a list of words where participants are required to respond whether the word was previously shown or not.


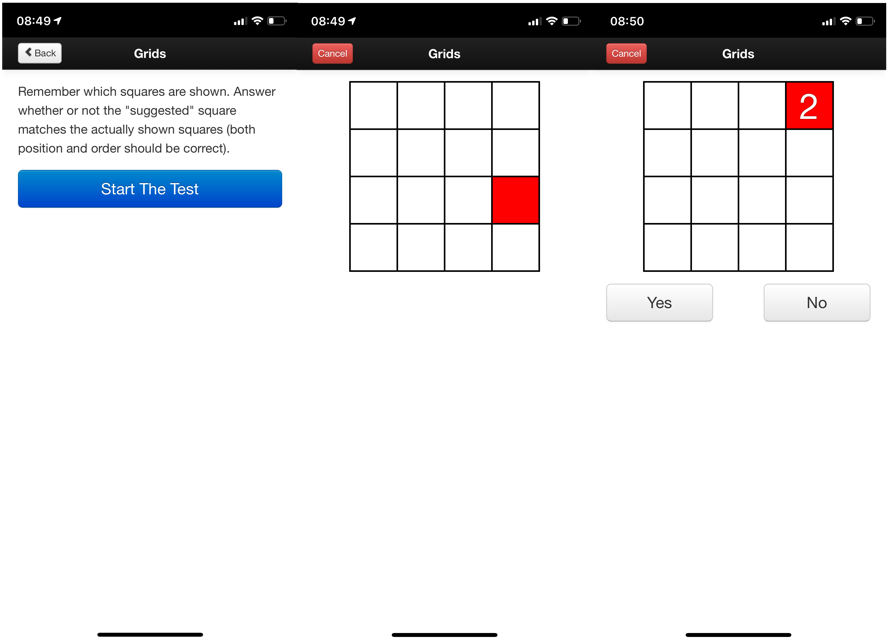


Figure S4. Images taken from the working memory test. Left shows the instructions given to participant. Middle shows one step in a sequence of red grid segments of which participants were asked to remember the order. Right poses the question of whether this given red segment was presented second in the sequence.


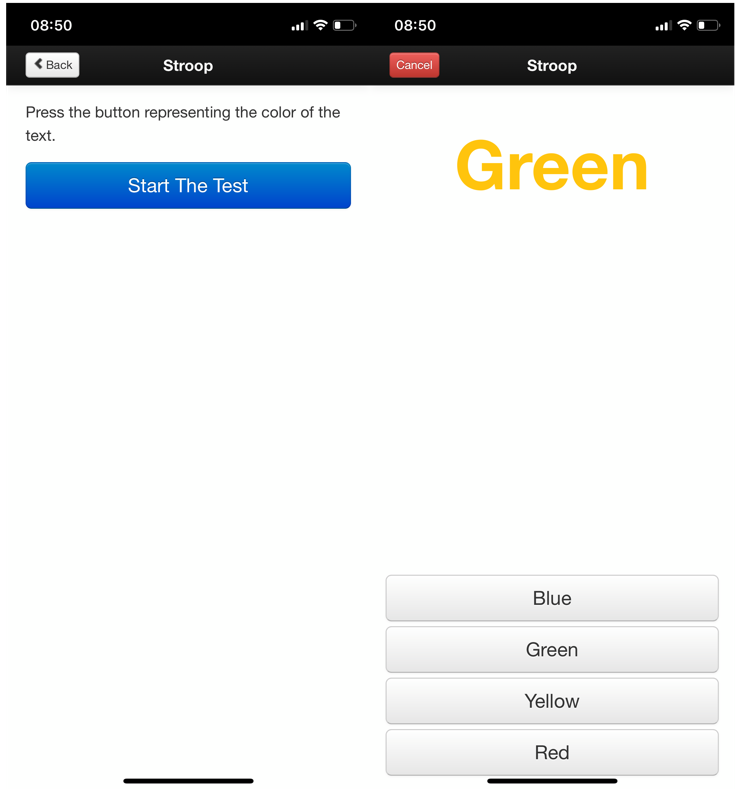


Figure S5. Images taken from the Stroop test. Left shows the instructions given to participant. Right shows an example of colour-text stimulus and buttons by which participants respond.


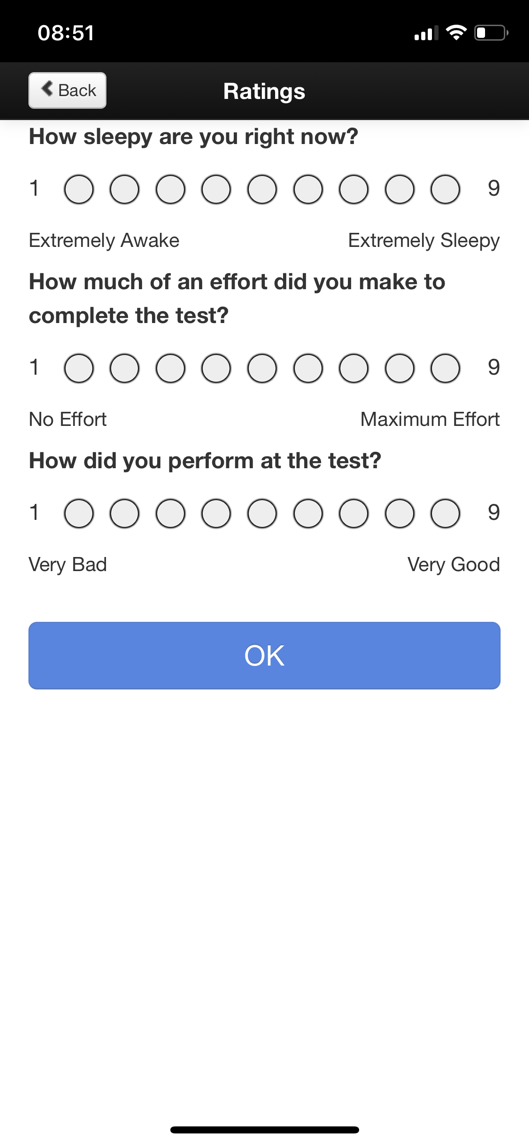


Figure S6. Image of subjective ratings provided by participant following each cognitive test.

Figure S7. Histograms of all participants’ responses times split by test. S7A: Simple attention, S7B: Arithmetic, S7C: Working memory, S7D: Stroop.
